# Oral nano-delivery of anticancer ginsenoside 25-OCH_3_-PPD, a natural inhibitor of the MDM2 oncogene: Nanoparticle preparation, characterization, *in vitro* and *in vivo* anti-prostate cancer activity, and mechanisms of action

**DOI:** 10.18632/oncotarget.4091

**Published:** 2015-05-24

**Authors:** Sukesh Voruganti, Jiang-Jiang Qin, Sushanta Sarkar, Subhasree Nag, Ismail A. Walbi, Shu Wang, Yuqing Zhao, Wei Wang, Ruiwen Zhang

**Affiliations:** ^1^ Department of Pharmaceutical Sciences, Texas Tech University Health Sciences Center, Amarillo, TX 79106, USA; ^2^ Cancer Biology Center, School of Pharmacy, Texas Tech University Health Sciences Center, Amarillo, TX 79106, USA; ^3^ Nutritional Science Program, Texas Tech University, Lubbock, TX 79409, USA; ^4^ School of Traditional Chinese Materia Medica, Shenyang Pharmaceutical University, Shenyang 110016, China

**Keywords:** molecular targeting efficiency, MDM2, ginsenoside, PEG-PLGA nanoparticles, oral delivery

## Abstract

The Mouse Double Minute 2 (*MDM2)* oncogene plays a critical role in cancer development and progression through p53-dependent and p53-independent mechanisms. Both natural and synthetic MDM2 inhibitors have been shown anticancer activity against several human cancers. We have recently identified a novel ginsenoside, 25-OCH_3_-PPD (GS25), one of the most active anticancer ginsenosides discovered thus far, and have demonstrated its MDM2 inhibition and anticancer activity in various human cancer models, including prostate cancer. However, the oral bioavailability of GS25 is limited, which hampers its further development as an oral anticancer agent. The present study was designed to develop a novel nanoparticle formulation for oral delivery of GS25. After GS25 was successfully encapsulated into PEG-PLGA nanoparticles (GS25NP) and its physicochemical properties were characterized, the efficiency of MDM2 targeting, anticancer efficacy, pharmacokinetics, and safety were evaluated in *in vitro* and *in vivo* models of human prostate cancer. Our results indicated that, compared with the unencapsulated GS25, GS25NP demonstrated better MDM2 inhibition, improved oral bioavailability and enhanced *in vitro* and *in vivo* activities. In conclusion, the validated nano-formulation for GS25 oral delivery improves its molecular targeting, oral bioavailability and anticancer efficacy, providing a basis for further development of GS25 as a novel agent for cancer therapy and prevention.

## INTRODUCTION

The majority of current cancer chemotherapeutic agents are natural product derivatives, and natural products represent valuable sources of bioactive compounds, with many naturally-occurring compounds and their synthetic analogs being developed for cancer therapy and prevention in both preclinical and clinical settings [1–2]. One such natural product with a long history of chemopreventive usage is ginseng, which has been used for the treatment and prevention of many diseases, including cancer [3–4]. The anticancer properties of ginseng have largely been attributed to its saponin constituents, which are termed ginsenosides [4]. We have recently identified a novel ginsenoside, 25-OCH_3_-PPD (GS25), from *Panax notoginseng*, which is thought to be the most potent anticancer ginsenoside discovered thus far [5–6]. GS25 has been shown to be active against several human cancers such as lung [7], pancreatic [8], breast [9], and prostate cancers [10–11]. In various cancer cell lines, GS25 inhibited proliferation, induced cell cycle arrest and apoptosis, and inhibited cell migration *in vitro*, and exerted these effects while also preventing metastasis *in vivo* [9]. In addition, GS25 sensitized prostate cancer cells to chemotherapy and radiation therapy [10].

Our mechanistic studies have demonstrated that inhibition of the *MDM2* oncogene is one the major mechanisms responsible for the anticancer activity of GS25 [7–11]. The *MDM2* oncogene is amplified and/or overexpressed in many human cancers, including prostate cancer [12–14]. We and other investigators have demonstrated that MDM2 has both p53-dependent and -independent oncogenic activities; it is considered a promising molecule for developing targeted cancer therapy and prevention approaches [15–22]. Several MDM2 inhibitors under preclinical and clinical development have been shown to have excellent efficacy, including Nutlin-3 [23], RITA [24], MI-219 [25], SP-141 [26–27], and JapA [28], although their mechanisms of action vary. As a natural product-derived MDM2 inhibitor, GS25 has dual inhibitory functions, *i.e*., inhibiting *MDM2* transcription and inducing MDM2 protein autoubiquitination and degradation [9], which is different from the other reported MDM2 inhibitors. In addition, GS25 exerts its MDM2 inhibitory activity and anticancer effects in a p53-independent manner, which is critical, since more than half of human cancers have p53 mutations or dysfunctional p53.

GS25 is now under preclinical development as a novel anticancer agent. However, as seen with other natural compounds, its therapeutic applications are limited by low aqueous solubility and instability under harsh conditions, resulting in pharmacokinetic restrictions such as low bioavailability by oral administration, extensive metabolism, and rapid elimination [29]. An ideal solution to the bioavailability problem is to develop a formulation which protects the drug in its intact form and increases its absorption and bio-stability. Recently, a self-emulsifying drug delivery system (SEDDS) for GS25 was developed to allow oral administration, but there was no evidence of improved anticancer efficacy of the drug when it was administered in an emulsion [30]. Therefore, it is of high importance to develop an orally active formulation for GS25 that can provide improved anticancer efficacy and minimal toxicity.

Biodegradable polymeric nanoparticle-based drug delivery systems are extensively used to improve the bioavailability and enhance the efficacy of therapeutic drugs. Encapsulation of drugs with nanoparticles protects the molecules from premature degradation, increases their solubility, promotes controlled drug release, and improves drug targeting, often resulting in improved therapeutic efficacy [31–32]. Different materials, such as chitosan, cyclodextrins, polymers, and dendrimers have been employed as carriers to improve drug bioavailability [33–34]. Among them, Poly(lactic-co-glycolic acid) (PLGA) is an efficient carrier for the delivery of hydrophobic drugs and has been approved by the U.S. Food and Drug Administration (FDA) for use in therapeutic formulations due to its biodegradability and biocompatibility [35]. There is increasing evidence that PLGA can efficiently improve the aqueous solubility, permeability and bioavailability of many potent drugs that are difficult to deliver orally, such as curcumin and paclitaxel [35–37]. However, PLGA nanoparticles exhibit short circulation times due to their rapid clearance by cells of the mononuclear phagocytic system (MPS) [38]. Surface coating nanoparticles with hydrophilic polymers, such as polyethylene glycol (PEG), sterically stabilizes the particles, leading to increased plasma circulation and drug bioavailability, as well as a prolonged half-life, improving the drug targeting efficacy [39]. Therefore, in the present study, we designed and prepared GS25-loaded PEG-PLGA nanoparticles (GS25NP) in order to improve the oral bioavailability of GS25.

The specific goals of the present study were to design, prepare, and optimize the formulation for GS25 and to demonstrate that the new formulation increased the oral absorption and improved the anticancer efficacy at a low dose. The physicochemical and pharmacological properties of GS25NP were evaluated both *in vitro* and *in vivo*. In addition, a preliminary assessment of the safety profile of GS25NP was accomplished. The investigation described herein is highly significant, and is relevant to the development of GS25 as a therapeutic agent for cancer therapy and prevention.

## RESULTS

### Preparation and characterization of GS25-loaded PEG-PLGA nanoparticles (GS25NP)

**Figure 1:**
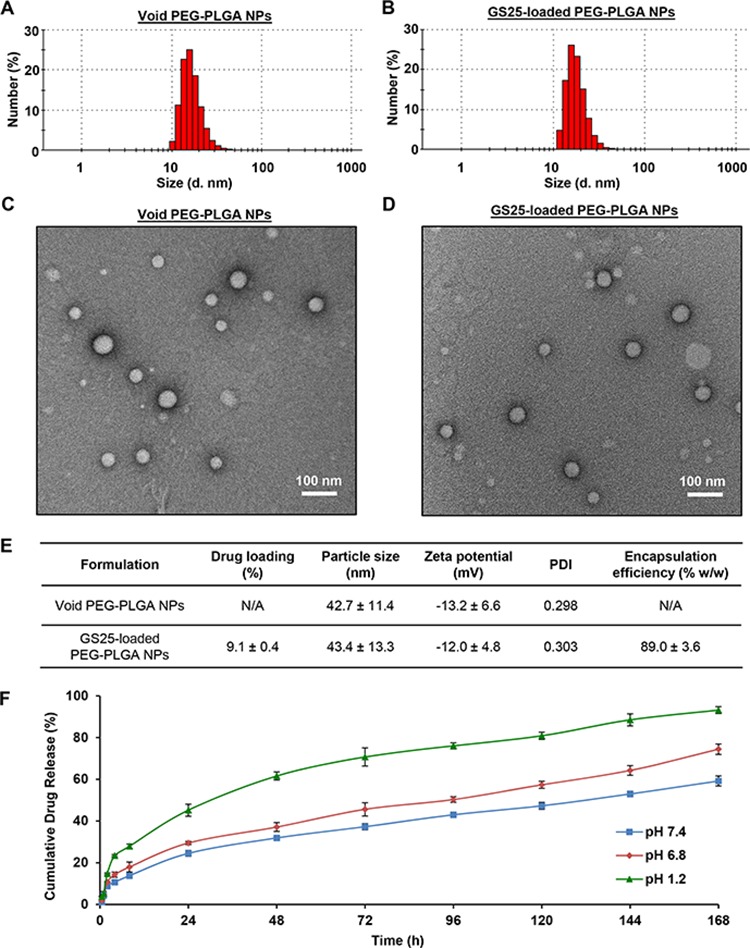
Preparation and characterization of PEG-PLGA nanoparticles The size and size distribution of the **A.** void and **B.** GS25-loaded PEG-PLGA NPs determined by dynamic light scattering. The morphology of the **C.** void and **D.** GS25-loaded PEG-PLGA NPs examined by transmission electron microscopy. **E.** The characteristics of the void and GS25-loaded PEG-PLGA NPs. PDI, polydispersity. **F.** The cumulative release kinetics of GS25 from GS25NP in simulated gastric fluid (pH 1.2), simulated intestinal fluid (pH 6.8) without enzymes and PBS (pH 7.4). The concentration unit for GS25NP is GS25 equivalent in all experiments and all experiments were repeated three times.

Both void PEG-PLGA nanoparticles (NPs) and GS25-loaded nanoparticles (GS25NP) were prepared, and representative diagrams showing the sizes and size distributions of the void NPs and GS25NP by dynamic light scattering (DLS) are presented in Figures 1A and 1B, respectively. The narrow, monomodal particle size distribution was further confirmed by transmission electron microscopy (TEM) (Figures 1C and 1D). Both DLS and TEM established that the average diameters of the void NPs and GS25NP were ~ 43 nm, with a low polydispersity (PDI = 0.3) (Figure 1E). The zeta potentials of the void NPs and GS25NP were measured to be −13.2 and −12.0 mV, respectively (Figure 1E). The drug loading and encapsulation efficiency of GS25NP was determined to be 9.1% (w/w) and 89% (w/w), respectively, using an established LC-MS/MS method [29].

The stability of GS25NP was assessed by measuring the cumulative release of GS25 at physiological pH condition (pH 7.4), in simulated intestinal fluid (pH 6.8), and in simulated gastric fluid (pH 1.2). Over an 8 h period, 13.8%, 18.0%, and 27.9% of the total GS25 was released from the nanoparticles at pH 7.4, pH 6.8, and pH 1.2, respectively, confirming that the majority of GS25NP is stable under these conditions (Figure 1F). The cumulative drug release after 24 h was found to be 24.5%, 29.5%, and 45.2%, respectively, at these pH values. There was a steady release of GS25 over the next six days (Figure 1F). This provides strong evidence that GS25NP is able to allow sustained release of the compound in both the stomach and intestine, protecting GS25 from premature degradation.

### *In vitro* permeability and cellular uptake of GS25NP

**Figure 2:**
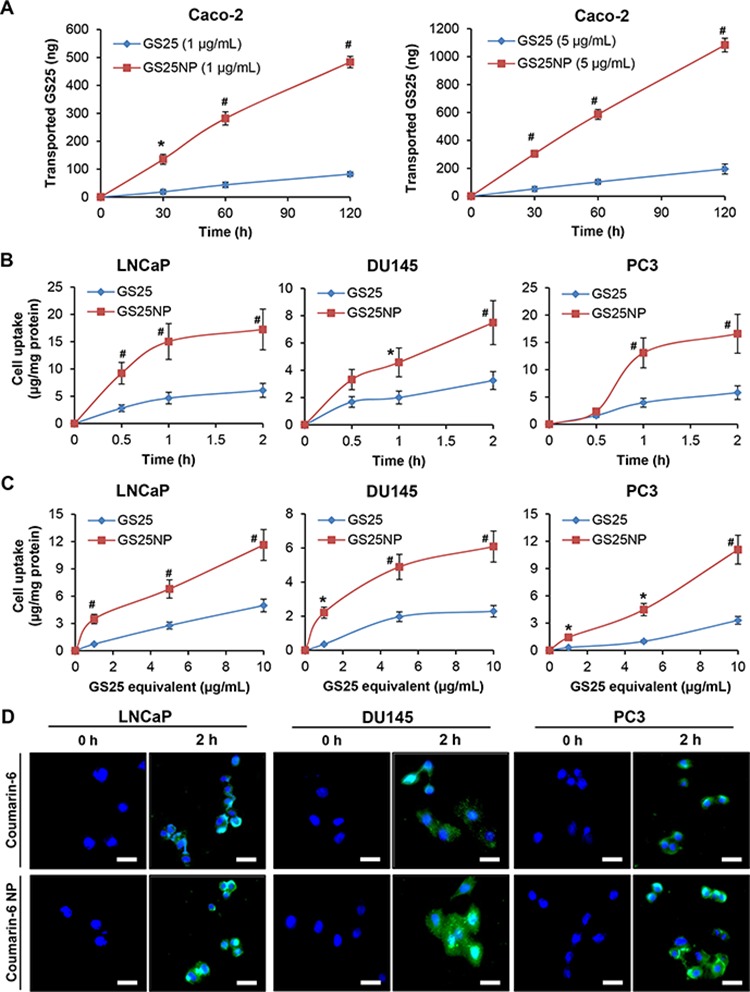
*In vitro* permeability and cellular uptake of GS25 and GS25NP **A.** The permeation of 1 and 5 μg/mL of free GS25 and GS25NP from apical to basolateral across Caco-2 cell monolayers at 37°C, presented as the amount of GS25 transported. **B, C.** The cellular uptake of GS25 and GS25NP in prostate cancer cells. Cells were treated with B 10 μg/mL of GS25 or GS25NP for 2 h or C 1, 5 or 10 μg/mL of GS25 or GS25NP for 1 h. GS25 was extracted and quantified by a LC-MS/MS analysis and normalized to the protein content. **D.** Cells were incubated with free coumarin-6 or coumarin-6-loaded NPs for 2 h, then the cellular uptake was monitored by a fluorescence microscope (scale bar, 20 μm). The concentration unit for GS25NP is GS25 equivalent in all experiments and all experiments were repeated three times. (*P < 0.05 and ^#^*P* < 0.01).

The effects of the encapsulation of GS25 into PEG-PLGA nanoparticles on the permeability of the drug were investigated using the Caco-2 cell line, a well-characterized model for intestinal epithelial permeability studies. As shown in Figure 2A, the transepithelial transport of GS25 was significantly enhanced by the nano-delivery system, in a time- and dose-dependent manner. After a 2 h incubation, there was an approximately 6-fold increase in GS25 transport in the nanoparticle groups with both low and high concentrations of GS25, compared to that for GS25 alone. The apparent permeability coefficients (*P*_app_) of GS25 and GS25NP were 3.8 × 10^−5^ cm/s and 24.6 × 10^−5^ cm/s, respectively, indicating that there was a carrier-mediated increase in the permeability of GS25. The transepithelial electrical resistance (TEER) was not significantly affected by GS25 and GS25NP throughout the experiment, which suggested that the increased GS25 transport was not due to a decrease in the monolayer integrity or the opening of tight junctions.

The cellular uptake of GS25 and GS25NP was subsequently investigated in the LNCaP, DU145, and PC3 prostate cancer cell lines. As shown in Figure 2B, the cellular uptake increased in a time-dependent manner for both GS25 and GS25NP. Compared to that of free GS25, there was an approximately three-fold high cellular uptake of GS25NP in all three cell lines after a 2-h incubation. Similarly, in the experiments using different doses of GS25, the uptake of the drug into cancer cells was also largely improved by the nano-delivery system (Figure 2C). Furthermore, the nanoparticle-induced enhancement of the cancer cell uptake of the encapsulated drug was confirmed using coumarin-6 and coumarin-6-loaded NPs. The fluorescence detection of this marker indicated that there was a significant increase in the uptake of this compound into all three prostate cancer cell lines (Figure 2D).

### *In vitro* cytotoxicity of GS25NP

**Figure 3:**
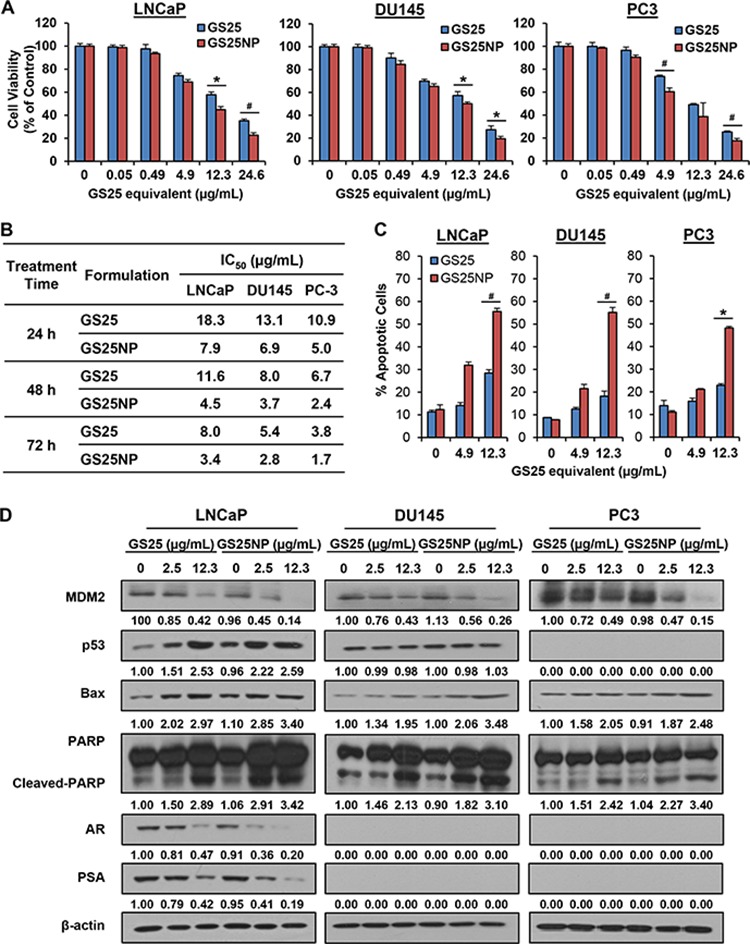
*In vitro* cytotoxicity of GS25 and GS25NP LNCaP, DU145, and PC3 cells were exposed to various concentrations of GS25 and GS25NP for 24, 48, or 72 h for determination of **A.** the cell viability (24 h) and **B.** IC_50_ values. The same cell lines were exposed to various concentrations of GS25 and GS25NP for **C.** 48 h for the determination of cell apoptosis, which was evaluated by the Annexin V-FITC method and **D.** for 24 h to determine the expression levels of various proteins by Western blotting. The intensity ratio under each band was obtained by IMAGEJ software analysis normalized on untreated control. The concentration unit for GS25NP is GS25 equivalent in all experiments and all experiments were repeated three times. (*P < 0.05 and ^#^*P* < 0.01).

Given the improved stability and cellular uptake of GS25 by the nano-formulation, we further compared the *in vitro* cytotoxicity of GS25 and GS25NP in prostate cancer cells. Considering that GS25 is a natural MDM2 inhibitor that can exert its anticancer activity in a p53-independent manner, three prostate cancer cell lines with different p53 backgrounds were selected for this study, *i.e*. LNCaP (p53 wild-type), DU145 (p53 mutant), and PC3 (p53 null). As shown in Figures 3A and 3B, the cell viability assays (24 h) for GS25 and GS25NP revealed that nanoparticle encapsulation of GS25 resulted in a ~ 50% decrease in the IC_50_ values compared with free GS25 in all three cell lines. Similar results were obtained after 48 and 72 h post-treatment, suggesting that there was sustained release of GS25 from the nanoparticles (Figure 3B).

We further compared the effects of GS25 and GS25NP on apoptosis in these prostate cancer cell lines. A significant enhancement of GS25-induced apoptosis by our delivery system was observed in all three cell lines (Figure 3C). At a concentration of 12.3 μg/mL, 48 h treatment with GS25NP resulted in about 55.6%, 55.2%, and 48.2% apoptotic cells in the LNCaP, DU145, and PC3 cell lines, respectively. However, only 28.5%, 18.2%, and 22.9% of the free GS25-treated cells underwent apoptosis. Consistent with the results for apoptosis, GS25NP decreased MDM2 protein level and increased the protein levels of the wild-type p53, Bax, and cleaved-PARP at a very low concentration (2.5 μg/mL), while the free GS25 did not produce significant effects on these apoptosis-associated proteins at this concentration (Figure 3D, compare intensity ratio of lane 2 with that of lane 5 in each band). Similar results were observed with regard to the inhibitory effects of GS25 and GS25NP on the expression of the androgen receptor (AR) and prostate-specific antigen (PSA) in LNCaP cells.

### *In vivo* pharmacokinetics of GS25NP

**Figure 4:**
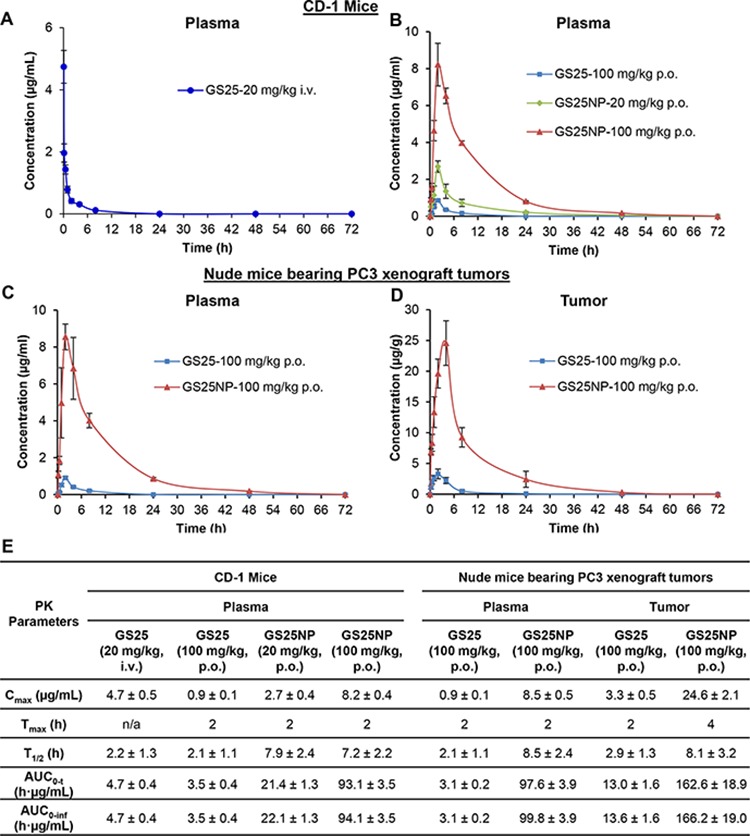
Pharmacokinetics and biodistribution of GS25 and GS25NP The plasma concentration-time curves following **A.** intravenous administration of 20 mg/kg GS25 or **B.** oral administration of GS25 (100 mg/kg) or GS25NP (20 and 100 mg/kg) to CD-1 mice. **C.** The plasma concentration-time curves following oral administration of GS25 (100 mg/kg) or GS25NP (100 mg/kg) to nude mice bearing PC3 xenograft tumors. **D.** The time-dependent distribution of GS25 and GS25NP in PC3 xenograft tumors. **E.** The pharmacokinetic parameters of GS25 and GS25NP in CD-1 mice and nude mice bearing PC3 tumors. The concentration unit for GS25NP is GS25 equivalent in all experiments.

The *in vivo* pharmacokinetics of GS25 and GS25NP were first assessed in male CD-1 mice. As shown in Figures 4A and 4B, compared to the i.v. injection of 20 mg/kg GS25, oral administration of GS25 (100 mg/kg) caused a very low peak value of drug concentration (*C*_max_ = 0.9 μg/mL) due to its low bioavailability (~ 14.9%). The nanoparticle encapsulation of GS25 resulted in a dramatically altered plasma concentration–time profile, compared with that of free GS25. However, compared to the oral administration of GS25 at the dose of 100 mg/kg, the *C*_max_ values for the oral administration of 20 and 100 mg/kg GS25NP were 3- and 9-fold higher, respectively, indicating that the absorption of GS25 was greatly increased after it was encapsulated into the nanoparticles. Similarly, compared to the oral administration of GS25 (T_1/2_ = 2.1 h), the half-life for the oral administration of GS25NP was extended to more than 7 h. Further, the AUC_*0-t*_ values for the 20 and 100 mg/kg oral doses of GS25NP increased to 21.4 and 93.1 h · μg/mL, compared with that of the 100 mg/kg oral GS25 (3.5 h · μg/mL), which suggested that the encapsulation greatly improved the oral bioavailability of GS25.

To further investigate the effects of nanoparticle encapsulation on the tumor uptake of GS25 *in vivo*, pharmacokinetic studies of GS25 and GS25NP were performed in nude mice bearing PC3 xenograft tumors. Consistent with the pharmacokinetic profiles in CD-1 mice, encapsulation of GS25 into nanoparticles increased the *C*_max_ and prolonged the half-life in the plasma of nude mice, indicating no significant strain-related variations (Figure 4C). More importantly, there was a large increase in the tumor uptake of GS25NP compared to GS25 alone (Figure 4D). The *C*_max_ was approximately 8-fold higher for GS25NP compared to GS25, and both the half-life and T_max_ were extended, which allowed for better tumor targeting of GS25NP than free GS25 *in vivo*. Selected pharmacokinetic parameters for GS25 and GS25NP in both mouse models are shown in Figure 4E.

**Figure 5:**
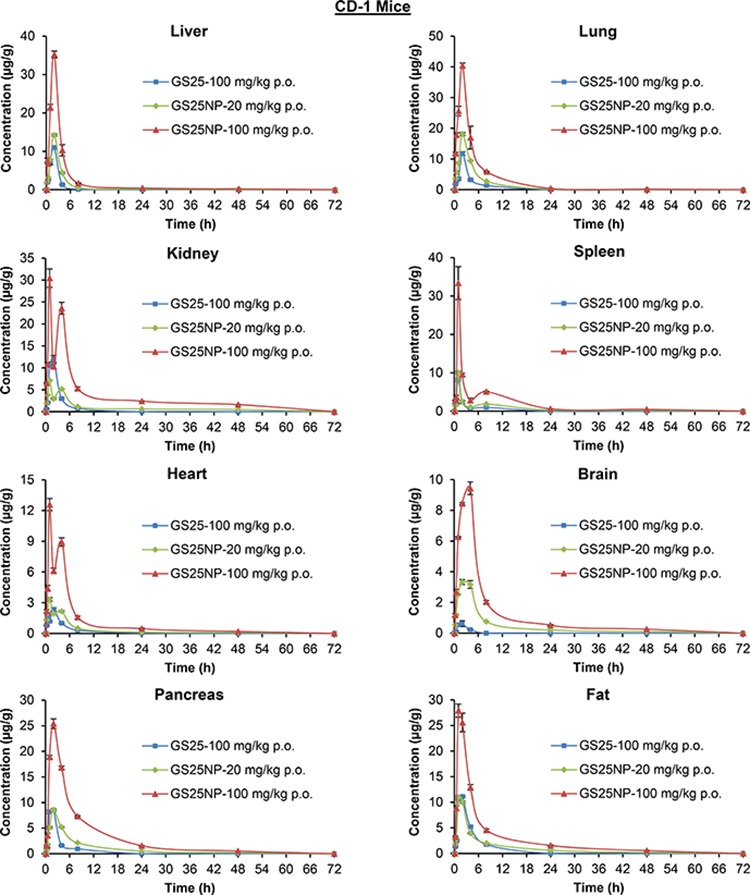
Tissue biodistribution of GS25 and GS25NP The time-dependent distribution of GS25 and GS25NP in various tissues (liver, lungs, kidneys, spleen, heart, brain, pancreas, and fat) of CD-1 mice after an oral dose of 100 mg/kg of GS25, 20 mg/kg of GS25NP, or 100 mg/kg of GS25NP. The concentration unit for GS25NP is GS25 equivalent in all experiments.

**Figure 6:**
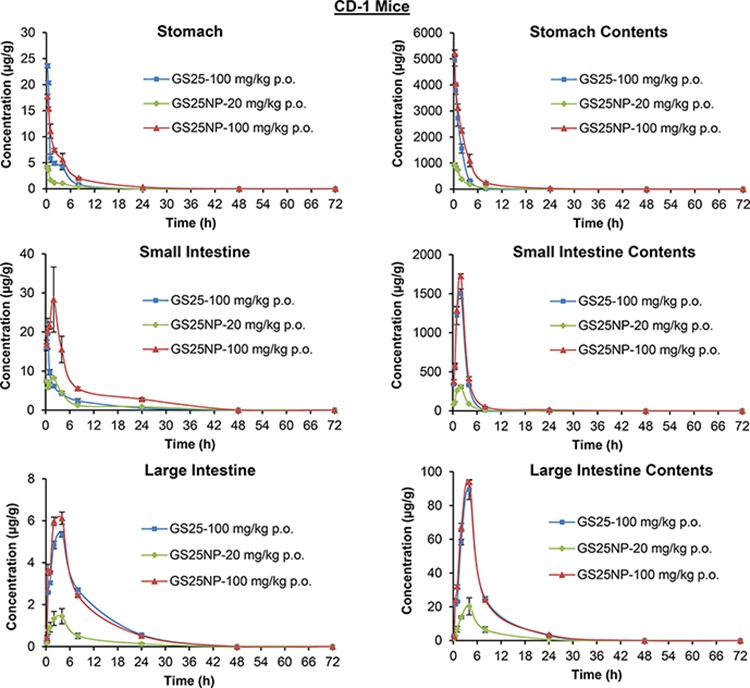
Biodistribution of GS25 and GS25NP in the gastrointestinal tract The time-dependent distribution of GS25 in the gastrointestinal tract of CD-1 mice after an oral dose of 100 mg/kg of GS25, 20 mg/kg of GS25NP, or 100 mg/kg of GS25NP. The concentration unit for GS25NP is GS25 equivalent in all experiments.

The tissue biodistribution of GS25 and GS25NP was also analyzed; the drug accumulation was increased by the nano-delivery system in almost all of the tissues tested, including liver, lungs, kidneys, spleen, heart, brain, pancreas, and fat (Figure 5), except the gastrointestinal (GI) tract (Figure 6). Although GS25NP had better stability under physiological conditions, the drug concentrations for GS25 (100 mg/kg) and GS25NP (100 mg/kg) in the GI tract were almost the same (Figure 6), which further confirmed the better absorption of GS25NP in the GI tract.

### *In vivo* efficacy and toxicity of GS25NP

**Figure 7:**
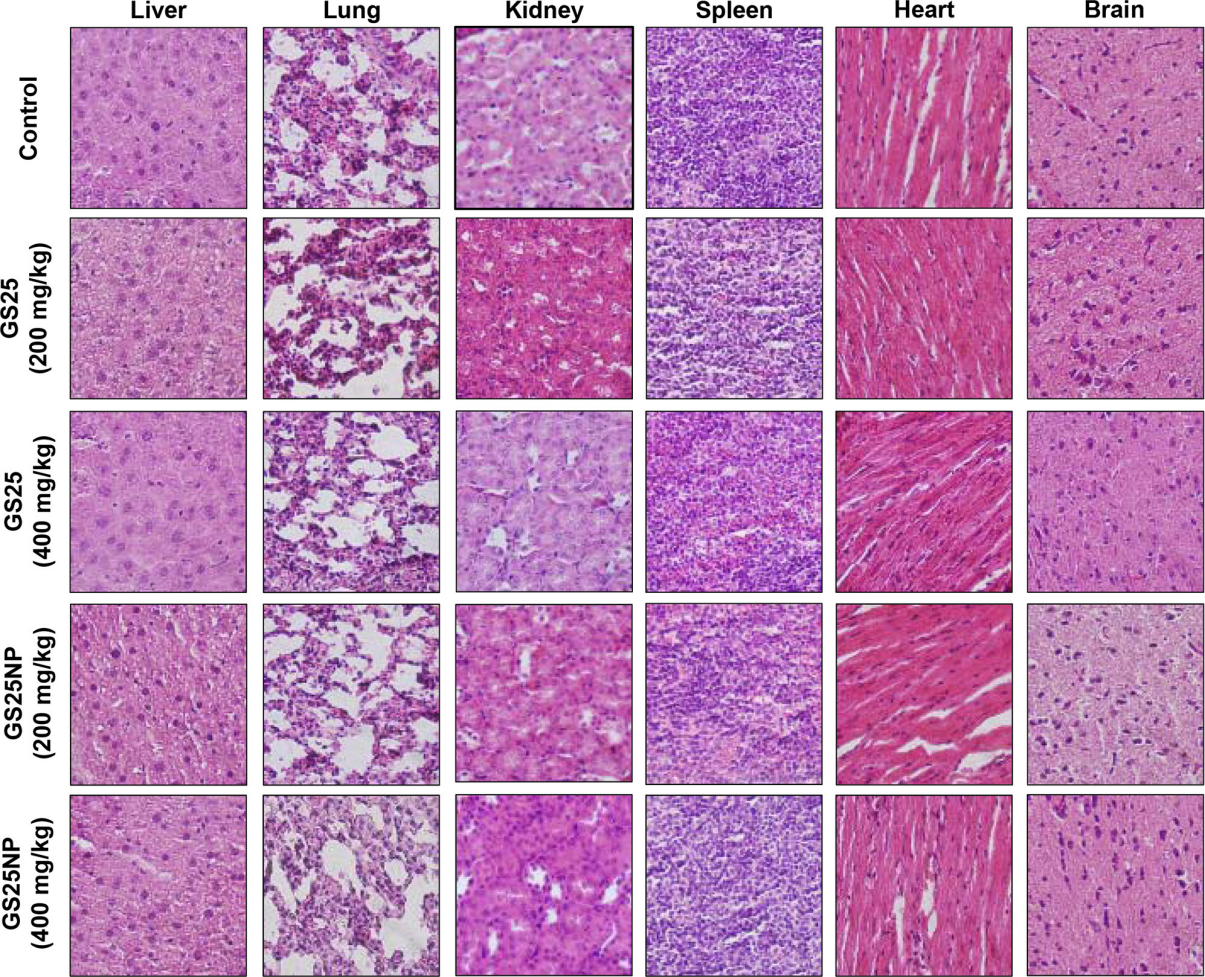
No host toxicity was caused by GS25 and GS25NP CD-1 mice were orally treated with GS25 or GS25NP at a dose of 200 or 400 mg/kg/d for 7 days. At the end of the experiments, H&E staining of the paraffin sections of various tissues (liver, lungs, kidneys, spleen, heart, and brain) from mice was performed. All images represented the series of sections. The concentration unit for GS25NP is GS25 equivalent in all experiments.

To determine the optimal therapeutic doses of GS25 and GS25NP for *in vivo* efficacy studies, initial maximum tolerated dose (MTD) studies were performed in CD-1 mice. The results showed no toxicity to the mice at a dose of up to 400 mg/kg of GS25 or GS25NP. No significant decrease in mouse body weight or any other signs of toxicity were observed. Further, a histological examination of the various tissues (liver, lungs, kidneys, spleen, heart, and brain) from mice treated with the vehicle, GS25, or GS25NP showed no significant differences among the groups (Figure 7).

**Figure 8:**
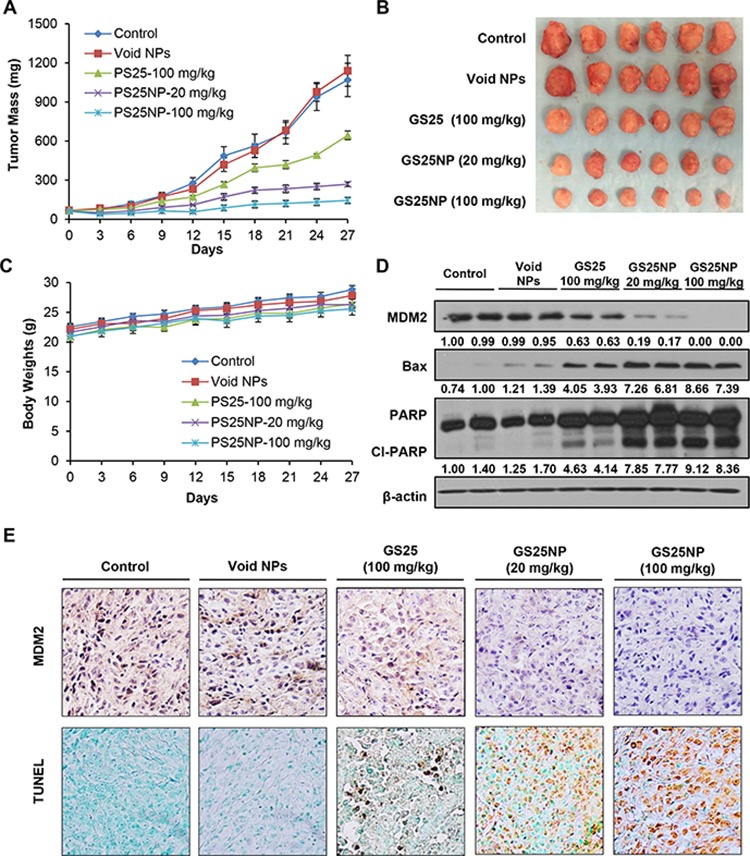
*In vivo* anticancer efficacy of GS25 and GS25NP **A.** Nude mice bearing PC3 xenograft tumors were treated with GS25 (100 mg/kg) or GS25NP (20 and 100 mg/kg) by oral administration 5 days/week for 4 weeks. The control mice received vehicle only or void nanoparticles. **B.** At the end of the experiments, representative tumors were removed and photographed. **C.** All mice were monitored for changes in body weight as a surrogate marker of toxicity. Tumors were excised and cut into multiple sections for **D.** Western blotting for the protein expression of MDM2, Bax and PARP, where the intensity ratio under each band was obtained by IMAGEJ software analysis normalized on untreated control; and **E.** MDM2 immunohistochemical staining and TUNEL staining. The concentration unit for GS25NP is GS25 equivalent in all experiments.

The anticancer efficacy of GS25 and GS25NP was evaluated using the PC3 xenograft model of human prostate cancer. As shown in Figures 8A and 8B, 100 mg/kg of GS25 had moderate effects on tumor growth, and a 4-week oral treatment led to approximately 41% inhibition of the PC3 tumor growth. However, treatment for the same period of time using 20 and 100 mg/kg of GS25NP inhibited the growth of PC3 xenograft tumors by approximately 75% and 87%, respectively (Figures 8A and 8B). In addition, there were no significant changes in the average body weights of the mice in any of the treatment groups, suggesting that the treatment did not lead to host toxicity (Figure 8C).

**Figure 9:**
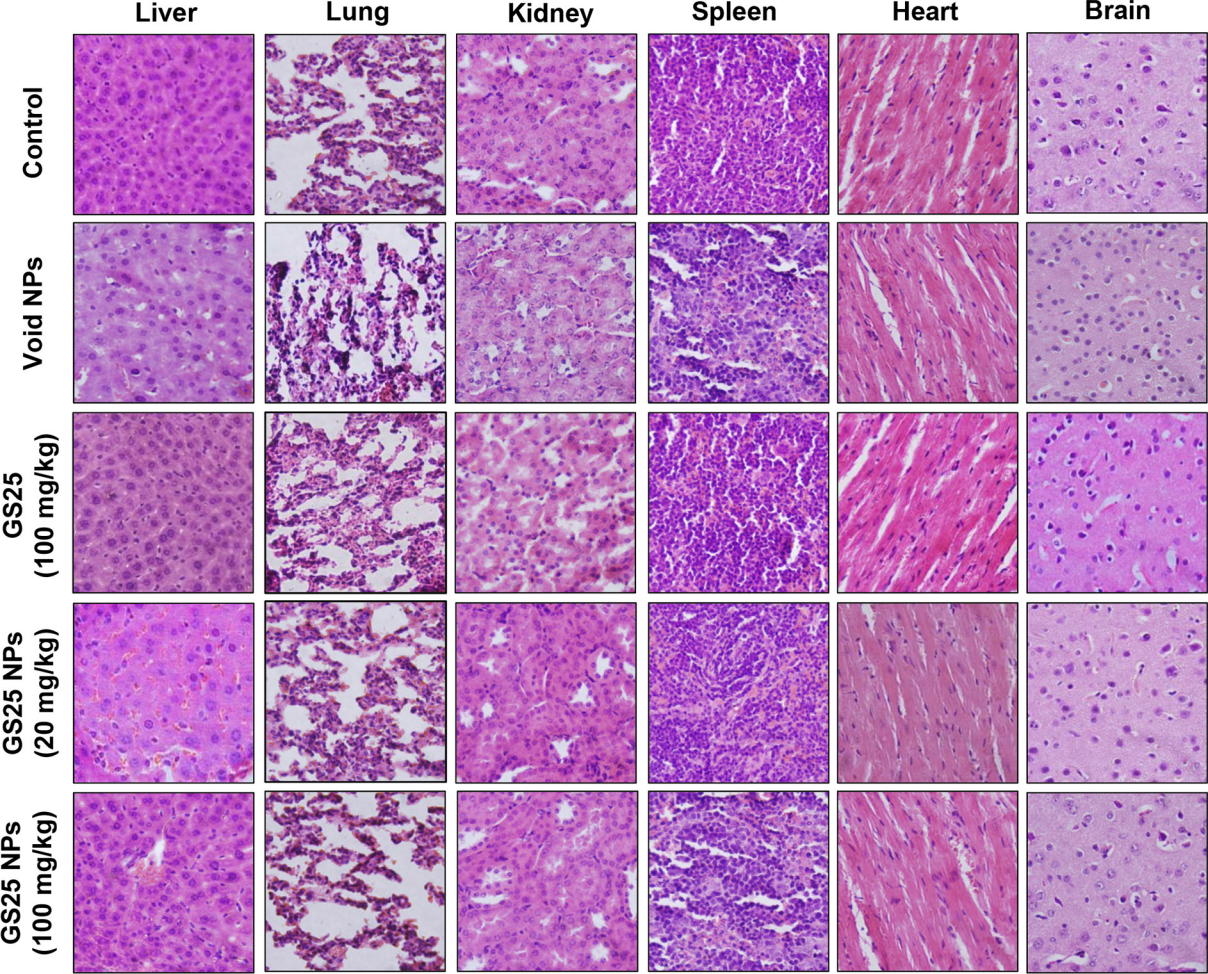
No host toxicity was caused by GS25 and GS25NP GS25 (100 mg/kg/day) and GS25NP (20 and 100 mg/kg/day) were administered orally to nude mice bearing PC3 xenografts 5 days/week for 4 weeks. At the end of experiments, H&E staining of the paraffin sections of various tissues (liver, lungs, kidneys, spleen, heart, and brain) from mice was performed. All images represented the series of sections. The concentration unit for GS25NP is GS25 equivalent in all experiments.

We further examined the expression levels of MDM2 and other apoptosis-related proteins *in vivo*. Consistent with the *in vitro* observations, nanoparticle encapsulation also enhanced the effects of GS25 on the expression of MDM2, Bax, and PARP *in vivo* (Figure 8D). The *in vivo* inhibition of MDM2 and increase in apoptosis in tumor tissues were further confirmed by an immunohistochemical analysis (Figure 8E). In addition, there were no significant differences in the histological findings between the control and various treatment groups in any of the tissues examined (liver, lungs, kidneys, spleen, heart, and brain), indicating that GS25NP treatment was safe at therapeutic doses (Figure 9).

## DISCUSSION

In the present study, we prepared and characterized a novel nano-delivery system for the oral administration of a natural MDM2 inhibitor, GS25, and evaluated its pharmacokinetics, toxicity, and efficacy in preclinical models of human prostate cancer. We have made several novel findings. First, an oral formulation of GS25 is developed by employing biodegradable PEG-PLGA copolymers, leading to the steady and sustained release of GS25. Second, the nanoparticle encapsulation of GS25 protects this drug from premature degradation in the stomach, increases its intestinal epithelial permeability, and improves its cancer cell uptake *in vitro* and tumor uptake *in vivo*. Third, this nanoparticle encapsulation dramatically alters the pharmacokinetic profiles of GS25, resulting in increased absorption, a prolonged half-life, and improved oral bioavailability. Fourth, GS25NP shows enhanced anticancer efficacy *in vitro* and *in vivo*, with initial effects beginning at low concentrations, suggesting that this nano-delivery system provides a remarkable dose advantage. Finally, GS25 does not induce toxicity in mice even at very high doses (up to 20-fold higher than the effective dose). These results demonstrate that our newly developed oral nano-formulation of GS25 improves its drug-like properties and enhances its anticancer efficacy without inducing toxicity, providing a basis for the further development of this drug for cancer treatment and prevention.

GS25 is a novel natural anticancer ginsenoside, and has shown therapeutic efficacy in preclinical models of several human cancers. There is an increasing interest in developing GS25 as an agent for cancer therapy and prevention due to its high efficacy and minimal toxicity. However, the potential clinical translation of GS25 has been limited by its poor bioavailability and short half-life, which stem from the low aqueous solubility, poor absorption, and instability under acidic conditions of this compound. Oral delivery is the most commonly used method of drug administration and it has a high level of patient acceptance. Therefore, an oral delivery system for GS25 is urgently needed for further development of this compound. Although a SEDDS formulation was developed for the oral administration of GS25, no evidence of the efficacy of this delivery system has been reported yet. Therefore, we have recently designed and developed an oral nano-formulation of GS25, which directly improves the bioavailability of the drug and significantly enhances its anticancer efficacy *in vitro* and *in vivo*.

It is known that the poor absorption and limited oral bioavailability of hydrophobic drugs, such as GS25, is mainly due to the unfavorable physicochemical properties as well as the GI mucus barriers [32]. Recently, various types of nanocarriers, including nanoparticles of biodegradable polymers are being developed to prevent the drug degradation caused by low pH and enzymes of the GI tract [40–42]. In the present study, to facilitate the transition of GS25 from preclinical to clinical studies, various FDA-approved nanoformulations, including PLGA have been considered as potential carriers for the oral delivery of this compound. Recent evidence demonstrates that PLGA is a safe and efficient nanosystem for the oral delivery of hydrophobic natural products, resulting in improved bioavailability and enhanced efficacy *in vitro* and *in vivo* [35–37]. Considering the acidic nature of PLGA monomers, we therefore designed and synthesized the PEG-PLGA nanoparticles for the oral delivery of GS25. Our initial studies showed that PEG-PLGA nanoparticles had high encapsulation efficiency of GS25 and a sustained release up to 1 week. It also stabilized the compound in the GI tract, increased plasma circulation, and prolonged half-life, leading to the improved drug targeting efficacy and bioavailability.

Although the tremendous nanotechnological advances have allowed many methods available for preparing nanoparticles, it is important to choose the best preparation method of nanoparticles according to the physicochemical properties of the drugs [40, 43–44]. Because GS25 is a liposoluble drug and has poor stability in the acidic environment, several methods that can be applied to this compound have been used to prepare PEG-PLGA nanoparticles, including emulsification-diffusion, solvent emulsion-evaporation, and nanoprecipitation. We then compared the nanoparticle size distribution, encapsulation efficiency, and the release kinetics of GS25-loaded nanoparticles obtained using different methods. Our results indicated that PEG-PLGA encapsulated GS25 successfully by nanoprecipitation method, having more than 9% drug loading and 89% encapsulation efficiency with an average particle size of 43 nm, which was in the same range as reported in other studies using the same nanoprecipitation method [44–46]. More importantly, the encapsulation of GS25 by PEG-PLGA nanoparticles improved its anticancer activity and inhibitory effects on MDM2, without disturbing its chemical structure.

In this study, we demonstrated that our novel oral nano-delivery system for GS25 allows it to exert substantial effects in a model of prostate cancer, suggesting that the compound has great potential for further development for use in the clinical setting. We first demonstrated that GS25NP has a gradual, steady, and sustained release profile under physiological conditions. It has been reported that once orally delivered, GS25 starts to be degraded in the stomach due to the acidic pH [29]. Our release kinetic studies in simulated physiological media have indicated that the majority of GS25NP is stable even under the harsh conditions of the stomach (pH 1.2) and small intestine (pH 6.8), resulting in an optimal release profile. Second, we demonstrated that nanoparticle encapsulation optimizes the absorption and bioavailability of GS25. Our studies using Caco-2 cell lines, CD-1 mice, and nude mice bearing PC-3 xenografts have shown that GS25NP has improved intestinal permeability, increased drug accumulation in plasma and various tissues, and an extended circulation lifetime, leading to optimal pharmacokinetic and biodistribution profiles.

Third, we demonstrated that GS25NP has better inhibitory effects on MDM2 *in vitro* and *in vivo*. Assays for the protein expression of MDM2 and other apoptosis regulators have indicated that GS25NP initiates its effects at a low dose, at which GS25 was not effective for inhibiting MDM2 either *in vitro* or *in vivo*. These results have been attributed to the increase in cancer cell uptake and tumor penetration of GS25NP. Fourth, we demonstrated that GS25NP has better *in vitro* and *in vivo* efficacy than free GS25. Our results have shown that GS25NP starts to inhibit prostate cancer cell viability at very low dose levels *in vitro* and *in vivo*, independent of the p53 status of the cells. These findings make it clear that this oral delivery system contributes to improving the efficacy of GS25 through various aspects, including the various factors mentioned above. Of note, our initial MTD studies indicated that GS25NP has a favorable safety profile and leads to no mortality even at a high dose of 400 mg/kg/d (administered for 7 days). No significant morphological changes were seen in various tissues obtained from these mice. Furthermore, we have found that there were no significant changes in the body weight or tissue morphology in nude mice bearing PC3 xenograft tumors after a 4-week treatment with GS25 (100 mg/kg) or GS25NP (20 and 100 mg/kg). However, further investigations on the long-term oral toxicity of GS25NP are needed.

In summary, we have prepared and characterized an oral nano-delivery system for a novel natural MDM2 inhibitor, GS25, and herein demonstrated its pharmacokinetics, efficacy, and safety in various preclinical models of human prostate cancer. These outcomes suggest that the newly developed oral formulation may have direct practical implications for developing GS25 as an agent for cancer therapy and prevention.

## MATERIALS AND METHODS

### Cell lines and cell culture

Human prostate cancer cell lines LNCaP (p53 wild-type), DU145 (p53 mutant), and PC3 (p53 null) and human intestinal epithelial cell line Caco-2 were purchased from the American Type Culture Collection (Rockville, MD, USA). LNCaP cells were cultured in RPMI 1640 medium. PC3 cells were grown in Ham's F-12 medium. DU145 and Caco-2 cells were cultured in Eagle's minimum essential medium (EMEM). All cell culture media contained 10% fetal bovine serum and 1% penicillin/streptomycin.

### PEG-PLGA polymers, chemicals, antibodies, and other reagents

The novel agent, 25-OCH_3_-PPD (GS25), was isolated and characterized as described in our earlier studies [5–6]. All chemicals and solvents were of the highest grade available. The m-polyethyleneglycol-polylactic-glycolic acid [mPEG (MW = 5,000 Da)-PLGA (50:50; MW = 45,000 Da)] polymers were purchased from Advanced Polymer Materials Inc. (Montreal, Canada). Acetone and poly-vinyl alcohol (PVA) were purchased from Sigma (St Louis, MO, USA). The anti-human p53, Bax, and PARP antibodies were from Santa Cruz Biotechnology Inc. (Dallas, TX, USA). The anti-human MDM2 antibody was from Calbiochem (Billerica, MA, USA). The anti-human PSA and AR antibodies were purchased from BD Pharmingen (San Diego, CA, USA).

### Preparation of PEG-PLGA nanoparticles

A total of 40 mg of PEG-PLGA polymers and 5 mg of GS25 were dissolved in 4 mL of acetone and then added drop-wise under rigorous mixing into 40 mL of DD H_2_O containing 0.5% PVA. The mixture was then sonicated for 5 min in a bath sonicator. The organic solvent in the mixture was evaporated by continuous stirring overnight. The resultant solution was centrifuged and washed with water twice to remove free drug, and the precipitate was resuspended in 5 mL of water. Finally, nanoparticle suspensions were freeze-dried for 48 h and stored at 4°C. Blank nanoparticles were produced in a similar manner without adding the drug.

### Analyses of the particle size, zeta potential, and morphology of PEG-PLGA nanoparticles

The size distribution and zeta potential of PEG-PLGA nanoparticles in water with 0.05% Tween 80 were determined using dynamic light scattering (Zetasizer 3000HS, Malvern Instruments Ltd, UK). The particle size was determined using a He-Ne laser beam at a wavelength of 633 nm with a fixed scattering angle of 90° at 25°C. The data were evaluated using the volume distribution. The zeta potential values were measured at the default parameters of the dielectric constant, refractive index, and viscosity of water, using a disposable capillary cell with a volume of 1 mL at 25°C. The morphology of the PEG-PLGA nanoparticles was examined by transmission electron microscopy (Hitachi H-9500, Hitachi High Technologies America, Inc. Dallas, TX, USA). Freeze-dried nanoparticles were dissolved in water with 0.05% Tween 80, and a small droplet was placed on a carbon-coated copper grid, followed by drying at room temperature before measurements were taken.

### Characterization of the drug loading and encapsulation efficiency of GS25NP

The drug loading and encapsulation efficiency of GS25NP were determined using the previously reported methods [47]. Briefly, 1 mg of freeze-dried GS25NP was dissolved in 1 mL of methanol and the mixture was incubated for 1 h in a 37°C water bath for complete extraction of GS25 into methanol. The solutions were centrifuged at 13,500 rpm for 5 min, and the amount of GS25 in the supernatant was determined using an LC-MS/MS method established in our previous study [29]. The drug loading and encapsulation efficiency were defined as the ratio of the amount of GS25 encapsulated in nanoparticles to the total amount of GS25NPs, and the ratio of the amount of encapsulated GS25 to that initially added in the process, respectively.

### *In vitro* release kinetic studies of GS25NP

The cumulative release of GS25 from GS25NP was studied in simulated gastric fluid (PBS adjusted to pH 1.2 with HCL), simulated intestinal fluid (PBS at pH 6.8) without enzymes and in PBS (pH 7.4). Briefly, 10 mg of GS25NP was dissolved in 1 mL of simulated fluid or PBS and sealed in a dialysis bag with a molecular weight cut-off of 10,000–13,000 Da. The dialysis bags were placed in 50 mL of release medium containing 0.05% Tween 80 at 37°C. The release medium (about 0.2 mL) was withdrawn at predetermined time intervals (15, 30, and 60 min, and 1, 2, 4, 8, 12, 24, 48, 72, 96, 120, 144, and 168 h). The collected samples were analyzed using the established LC-MS/MS method [29].

### Caco-2 cell monolayer permeability assay

The Caco-2 cell monolayer permeability assay was performed as reported previously [48]. In brief, Caco-2 cells were seeded onto polycarbonate 6-well Transwell^®^ inserts (mean pore size 3.0 μm, Corning Costar Inc., NY, USA) at a density of 4 × 10^5^ cells/well, then the confluent monolayers (10–12 days) were used for permeability studies. The transepithelial electrical resistance (TEER) of the monolayer was measured using an epithelial voltohmmeter (EVOM, WPI Inc., USA) to determine the formation of the monolayer and its integrity during the experiment. The studies were carried out in HBSS containing 30 mM HEPES at pH 6.0. Monolayers were washed with HBSS prior to the experiment, after which 0.5 and 1.2 mL of HBSS was placed into the upper and lower compartments, respectively. A total volume of 100 μL of solution (1 or 5 μg/mL) was taken from the lower compartment at regular intervals over 120 min and replaced with the same volume of fresh buffer, followed by a LC-MS/MS analysis. Apparent permeability coefficients (*P*_app_) were calculated using the equation: (∂Q/∂t)/(A*C_0_), where “∂Q/∂t” is the permeability rate of the drug across the cells, “*A*” is the diffusion area of the monolayer and “*C_0_*” is the initial concentration of GS25 in the upper compartment.

### Analysis of the cellular uptake of GS25NP

The uptake of GS25NP into prostate cancer cells was determined by a LC-MS/MS analysis and fluorescence detection [28–29]. Briefly, LNCaP, DU145, and PC3 cells were incubated with 10 μg/mL of GS25 or GS25NP for 0.5, 1, and 2 h or with 1, 5, and 10 μg/mL of GS25 or GS25NP for 1 h. Cells were then washed with cold PBS and kept at −80°C overnight for cell lysis. GS25 and cellular proteins were extracted by sonication for 1 min and centrifugation at 12,000 rpm for 10 min. The amount of GS25 in the cell lysates was quantified by a LC-MS/MS analysis and normalized to the protein content in each sample. For fluorescence detection, cells were incubated with coumarin-6 or coumarin-6-loaded NPs for 2 h and were fixed using 4% formalin in PBS. Cells were counterstained with DAPI and analyzed under an Olympus fluorescence microscope (Olympus America Inc).

### *In vitro* cytotoxicity studies

Assays for cell viability and apoptosis were performed as described previously [28, 49]. In brief, 3–4 × 10^4^ cells were seeded in a 96-well plate and treated with various concentrations of GS25 or GS25NP for 24, 48, or 72 h for the MTT assay. To detect apoptosis, 2–3 × 10^5^ cells were seeded in 6-well plates and were treated with GS25 or GS25NP for 48 h. Cells positive for Annexin V-FITC and PI were counted on a BD FACSVerse instrument (BD Biosciences, CA, USA).

### Western blotting analysis

In the *in vitro* studies, prostate cancer cells with or without GS25 or GS25NP treatment were lysed in NP40 lysis buffer containing a protease inhibitor mixture (Sigma, St. Louis, MO, USA). In the *in vivo* studies, tumor tissues were removed and homogenized in NP40 lysis buffer (100 mg tumor tissue/1 mL NP40 buffer), and the supernatants of the homogenates was collected. Then, the cell lysates and tumor homogenates were subjected to Western blotting analyses for the expression levels of MDM2 and other related proteins using the methods described in our previous studies [26–27, 50].

### Determination of the maximum tolerated dose (MTD)

To assess the possible host toxicity of GS25 and GS25NP and establish initial doses for *in vivo* treatment, a multi-dose MTD study was initially performed for both GS25 and GS25NP at a dose of 200 mg/kg for 7 days. Mice were monitored for mortality, body weight changes, and changes in physical appearance, with no signs of toxicity being observed at this dose. The toxicity studies were continued with dose of 400 mg/kg/d for 7 days using a similar protocol. At the end of the study, all mice were euthanized, and various tissues (liver, lungs, kidneys, spleen, heart, and brain) were collected for a pathological analysis.

### Determination of the *in vivo* efficacy of GS25NP in a prostate cancer xenograft model

The animal study protocol was approved by the Institutional Animal Use and Care Committee of the Texas Tech University Health Sciences Center. Male athymic pathogen-free nude mice (nu/nu, 4–6 weeks) were purchased from Charles River Laboratories (Wilmington, MA). The PC3 human prostate cancer xenografts were established as reported previously [26–27, 50]. Briefly, a total of 5 × 10^6^ PC3 cells (in 0.1 mL) were subcutaneously injected into the left inguinal area of the mice. All animals were monitored for activity, physical condition, body weight, and tumor growth. When the tumor volume reached ~ 100 mm^3^, the mice bearing PC3 xenografts were randomly divided into treatment and control groups (10 mice/each group). GS25NP was dissolved in water and administered by oral gavage at a dose of 20 mg/kg/d or 100 mg/kg/d, 5 day/week for 4 weeks, while GS25 in PEG400:EtOH:saline (57.1:14.3:28.6, v/v/v) was administered at a dose of 100 mg/kg/d for 4 weeks. The control groups received vehicle only or void NPs. At the end of the experiment, xenograft tumors and various organs (liver, lungs, kidneys, spleen, heart, and brain) were excised and snap frozen for Western blotting, immunohistochemical studies, TUNEL assays, and hematoxylin and eosin staining.

### Pharmacokinetic and biodistribution studies of GS25NP

Pharmacokinetic studies were carried out in normal CD-1 mice and nude mice bearing PC3 xenograft tumors as described previously [51–52]. Briefly, male CD-1 mice were divided randomly into four groups with three mice per time point. One group received 20 mg/kg of GS25 in PEG400:EtOH:saline by intravenous injection into the tail vein. One group of mice received GS25 in PEG400:EtOH:saline (57.1:14.3:28.6, v/v/v) at a dose of 100 mg/kg by oral administration. Two groups received GS25NP at a dose of 20 mg/kg and 100 mg/kg by oral administration. Nude mice bearing PC3 xenografts were divided into two groups and given GS25 and GS25NP at a dose of 100 mg/kg by oral administration. For orally-dosed mice, the plasma and various tissues (tumor, liver, lungs, kidneys, spleen, heart, brain, pancreas, fat, stomach, stomach contents, small intestine, small intestine contents, large intestine, and large intestine contents) were collected at 0, 15, 30, and 60 min, and 2, 4, 8, 24, 48, and 72 h after treatment. For mice that received an i.v. injection, various tissues were collected at 0, 5, 10, 30, and 60 min, and 2, 4, 8, 24, 48, and 72 h after treament. The plasma and tissue samples were stored at −80°C until they were analyzed by LC-MS/MS.

### Immunohistochemistry, TUNEL assay, and pathological analysis

All of the staining assays were performed as described previously [50]. Briefly, tissues were fixed and embedded in paraffin, cut into 5 μm sections, and then affixed onto glass slides. For the immunohistochemical studies, the tumor sections were blocked and incubated with a biotinylated anti-human MDM2 antibody (diluted 1:50 in 5% BSA in PBS) for 1–2 h at room temperature. Sections were then incubated with pre-diluted streptavidin-peroxidase HRP conjugates and stained with DAB chromogen according to the manufacturer's instructions. Finally, sections were lightly counterstained with hematoxylin. To detect apoptosis, tumor sections were stained with a TdT-mediated dUTP-biotin nick end labeling (TUNEL)-based In Situ Apoptosis Detection kit (Trevigen, Inc, Gaithersburg, MD) according to the manufacturer's instructions. For hematoxylin and eosin staining, the paraffin-embedded tissue sections were deparaffinized and stained with hematoxylin for 10 minutes and eosin for 1 minute. Tissue sections were analyzed under a phase-contrast Olympus microscope (Olympus America Inc).

### Statistical analysis

The data are expressed as the means ± SEM from at least three independent experiments. Two-sided *t*-tests were used for comparisons between two groups. A value of *P* < 0.05 was considered to be statistically significant.
